# Associations between fluid biomarkers and PET imaging ([11C]UCB‐J) of synaptic pathology in Alzheimer's disease

**DOI:** 10.1002/alz.70403

**Published:** 2025-07-10

**Authors:** Johanna Nilsson, Adam P. Mecca, Nicholas J. Ashton, Elaheh Salardini, Ryan S. O'Dell, Richard E. Carson, Andrea L. Benedet, Kaj Blennow, Henrik Zetterberg, Christopher H. van Dyck, Ann Brinkmalm

**Affiliations:** ^1^ Institute of Neuroscience and Physiology the Sahlgrenska Academy at the University of Gothenburg Gothenburg Sweden; ^2^ Yale Alzheimer's Disease Research Unit, Yale School of Medicine New Haven USA; ^3^ Wallenberg Centre for Molecular and Transla Medicine University of Gothenburg Gothenburg Sweden; ^4^ Department of Old Age Psychiatry Maurice Wohl Clinical Neuroscience Institute, King's College London London UK; ^5^ NIHR Biomedical Research Centre for Mental Health & Biomedical Research Unit for Dementia at South London & Maudsley NHS Foundation London UK; ^6^ Department of Radiology and Biomedical Imaging Yale University School of Medicine New Haven USA; ^7^ Department of Neurology & Neurosurgery McGill University Montréal Rue University Canada; ^8^ Clinical Neurochemistry Laboratory Sahlgrenska University Hospital Biskopsbogatan Mölndal Sweden; ^9^ UK Dementia Research Institute at UCL London UK; ^10^ Department of Neurodegenerative Disease UCL Institute of Neurology London UK; ^11^ Hong Kong Center for Neurodegenerative Diseases Hong Kong China

**Keywords:** Alzheimer's disease, biomarkers, mass spectrometry, SV2A, synaptic pathology

## Abstract

**INTRODUCTION:**

Positron emission tomography (PET) imaging with ligands for synaptic vesicle glycoprotein 2A (SV2A) has emerged as a promising methodology for measuring synaptic density in Alzheimer's disease (AD). We investigated the relationship between SV2A PET and CSF synaptic protein changes of AD patients.

**METHOD:**

Twenty‐one participants with early AD and seven cognitively normal (CN) individuals underwent [^11^C]UCB‐J PET. We used mass spectrometry to measure a panel of synaptic proteins in cerebrospinal fluid (CSF).

**RESULTS:**

In the AD group, higher levels of syntaxin‐7 and PEBP‐1 were associated with lower global synaptic density. In the total sample, lower global synaptic density was associated with higher levels of AP2B1, neurogranin, γ‐synuclein, GDI‐1, PEBP‐1, syntaxin‐1B, and syntaxin‐7 but not with the levels of the neuronal pentraxins or 14‐3‐3 zeta/delta.

**CONCLUSION:**

Reductions of synaptic density found in AD compared to CN participants using [^11^C]UCB‐J PET were observed to be associated with CSF biomarker levels of synaptic proteins.

**Highlights:**

- A panel of synaptic proteins was quantified in the CSF using mass spectrometry.
- SV2A ([^11^C]UCB‐J) PET was used to quantify synaptic density.
- Reductions of synaptic density were associated with CSF synaptic biomarker levels.

## 1 BACKGROUND

Synaptic dysfunction and degeneration are an early and significant part of the pathology occurring in Alzheimer's disease (AD) and in other neurodegenerative diseases.^1^ *Post mortem* brain studies have described a widespread reduction in synapse numbers in AD in comparison to healthy individuals, and this synaptic loss is the major structural correlate of cognitive decline, even more so than amyloid beta (Aβ) plaque pathology.^2^ These findings have contributed to an increasing interest in the development and implementation of synaptic biomarkers in diagnostics as well as mechanistic insight into synaptic pathology.

As early as 30 years ago, the first studies emerged verifying the presence of synaptic proteins in the cerebrospinal fluid (CSF). Since then, several methods for the quantification of a wide range of synaptic proteins in living patients have been developed as synaptic biomarkers.^3^ One of the most established synaptic biomarkers is neurogranin, a postsynaptic protein that is specifically increased in AD with changes at early stages of cognitive impairment^4^ and even before symptom onset.^5^ Other synaptic proteins, including SNAP‐25, synaptotagmin‐1, and GAP43, increase along with biomarkers of cerebral amyloid pathology in symptomatic and asymptomatic disease and are considered AD specific.^6^, ^7^, ^8^ Interestingly, some synaptic proteins are lower in CSF samples of participants with neurodegenerative disease. For example, neuronal pentraxins have decreased levels across symptomatic neurodegenerative diseases, including AD, compared to healthy individuals, and the levels of pentraxins strongly correlate with cognitive decline.^9^, ^10^, ^11^ Emerging evidence thus points to the fact that synaptic pathology mechanisms are more complex than had been previously believed and that all synaptic proteins may not represent the same pathological pathways.

Recent progress in synaptic positron emission tomography (PET) imaging has also allowed for the evaluation of synaptic alterations in vivo.^1^, ^12^ Utilizing [^11^C]UCB‐J, a PET tracer that binds the synaptic vesicle glycoprotein 2A (SV2A), which is expressed in nearly all synapses, the synaptic density of patients with AD was found to be reduced in the medial temporal and neocortical brain regions compared with healthy controls.^12^, ^13^ SV2A PET has been used to characterize the patterns of synaptic loss due to neurodegenerative disease, and early studies described the complex relationships with core AD biomarkers such as greater synaptic loss in areas of tau deposition,^14^ significant correlations between synaptic density and metabolism measured with [^18^F]Fludeoxyglucose ,^15^ and stage‐dependent correlations between hippocampal synaptic density and global amyloid measured with [^11^C]Pittsburgh Compound B ([^11^C]PiB).^16^ In addition, SV2A PET has been used to demonstrate significant synaptic loss in dementia with Lewy bodies,^17^ frontotemporal dementia,^18^ Parkinson's disease,^19^ and Huntington's disease.^20^ Thus, both imaging and CSF protein biomarkers are altered in AD and other neurodegenerative disorders, and these changes are likely to reflect synaptic pathology. However, the meaning of synaptic protein concentration changes measured in vivo with PET or CSF assays remains unclear. These changes may reflect altered clearance or altered protein production and secretion into the CSF due to synapse degeneration or changes in synaptic activity.^21^

In this study, we evaluated the relationship between SV2A PET and CSF synaptic protein changes of AD patients to gain insight into AD synaptic pathology and the meaning of synaptic protein biomarkers. To do so, we measured a panel of synaptic proteins in a small cohort of AD patients and control participants who have also undergone SV2A PET.

## 2 METHOD

### 2.1 Study design and population

The requirement of participation included fulfilling the diagnostic criteria for amnestic mild cognitive impairment (MCI) (*n* = 5)^22^ or probable dementia due to AD (*n* = 16).^23^ AD and MCI participants were additionally required to have a positive PET scan with [^11^C]PiB, a Mini‐Mental State Examination (MMSE) score < 26 and 24 to 30, respectively, and a Clinical Dementia Rating (CDR) score of 0.5 to 1.0 and 0.5, respectively. Cognitively normal (CN) participants (*n* = 7) were required to have a negative [^11^C]PiB PET scan, MMSE score < 26, and a CDR score of 0. All participants gave written informed consent approved by the Yale University Human Investigation Committee.

### 2.2 Brain imaging

To define regions of interest (ROIs) and perform partial volume correction (PVC) using the iterative Yang approach,^24^ T1‐weighted magnetic resonance imaging (MRI) was performed. FreeSurfer version 6.0 was used for volumetric segmentation and cortical reconstruction. A high‐resolution research tomograph (207 slices, resolution < 3 mm, full width at half maximum [FWHM]) was used for the PET scans with event‐by‐event motion correction. Dynamic [^11^C]PiB and [^11^C]UCB‐J scans 60 min were taken, following administration of a bolus of up to 555 and 740 MBq of tracer, respectively. As previously described, SRTM2 (cerebellum reference region) was used with dynamic scan data from 0 to 60 min to generate parametric images of [^11^C]PiB *BP*_ND_ and [^11^C]UCB‐J *DVR*.^24^

### 2.3 Liquid chromatography‐mass spectrometry analysis

CSF samples were collected in polypropylene tubes by lumbar puncture, centrifuged (2200 × g for 10 min, 20°C), and stored at −80°C. Eleven synaptic proteins were analyzed in the synaptic panel analysis; neurogranin, γ‐synuclein, the activating protein 2 subunit complex beta (AP2B1), rab GDP dissociation inhibitor alpha (GDI‐1), phosphatidylethanolamine‐binding protein 1 (PEBP‐1), 14‐3‐3 ζ/δ, syntaxin‐1B, syntaxin‐7, and the neuronal pentraxins (−1 [NPTX1], −2 [NPTX2], and the receptor [NPTXR]). The sample preparation of 100 µL of CSF samples entailed the addition of an internal standard (mix of stable‐isotope‐labeled peptides, 25 µL, 0.032 pmol/µL), reduction, alkylation, tryptic digestion, and solid‐phase extraction (for the complete protocol, refer to Nilsson et al.^25^). Quantification of the synaptic proteins was performed on a micro‐high‐performance liquid chromatography‐mass‐spectrometry system (6495 Triple Quadrupole LC/MS system, Agilent Technologies) equipped with a Hypersil Gold reverse‐phase C18 column (100 × 2.1 mm, particle size = 1.9 µm, Thermo Fisher Scientific) (for detailed settings, see Table S1). Assay performance was evaluated during the run by injections at regular intervals of a quality control sample.

**RESEARCH IN CONTEXT**

1. **Systematic review**: PET imaging with ligands for SV2A has emerged as a promising methodology for measuring synaptic density in AD. In parallel, numerous synaptic proteins have been identified as candidate biomarkers of synaptic degeneration in CSF. However, the meaning of synaptic protein concentration changes measured in vivo with PET or CSF assays remains unclear. We evaluate the relationship between SV2A PET and CSF synaptic protein changes in AD patients to gain insight into AD synaptic pathology and the meaning of synaptic protein biomarkers.
2. **Interpretation**: Our findings identify associations between reductions of synaptic density found in AD compared to CN participants using [11C]UCB‐J PET with CSF biomarker levels of synaptic proteins.
3. **Future directions**: Further studies in larger cohorts and a broader range of synaptic proteins may lead to a better understanding of the relationship between altered synaptic density fluid synaptic biomarkers in AD.

### 2.4 Data processing and statistical analysis

Skyline 20.1 (MacCoss Lab Software) was utilized for peak inspection and adjustment of the chromatographic spectra, and R software was used for the statistical analysis. For the proteins for which more than one peptide was analyzed, the peptide with the best repeatability (lowest coefficient of variation) was chosen for the statistical analysis (Table S2). The group comparisons of the demographic characteristics and biomarkers were evaluated by ANOVA and chi‐squared goodness‐of‐fit test for continuous and categorical variables, respectively. Associations between synaptic density and CSF biomarker levels were explored with Pearson's rank correlation analysis, and brain maps for visualization were created by setting each brain region's voxels uniformly to the calculated effect size (Pearson's *r*).

## 3 RESULTS

### 3.1 Participant characteristics and biomarker levels

The study sample was well balanced with respect to age and sex, and the AD participants had both typical clinical characteristics (MMSE = 23.0 ± 2.9) and core CSF biomarker levels of total tau (t‐tau), tau phosphorylated at threonine 181 (p‐tau181), Aβ42/40 (Aβ_42/40_) (Table 1). Out of the 11 synaptic proteins quantified, six proteins (neurogranin, γ‐synuclein, GDI‐1, PEBP‐1, 14‐3‐3 ζ/δ, and syntaxin‐1B) showed significantly higher protein levels (*p* < 0.05) in the AD group compared to the CN (Table 1).

**TABLE 1:** Demographics and biomarker characteristics.

	CN (*N* = 7)	AD (*N* = 21)	*p*
Sex, *n* (male, %)	5 (71.4%)	10 (47.6%)	0.274
Age (years)	73.4 (7.3)	69.1 (8.7)	0.246
MMSE score	28.9 (1.35)	23.0 (2.9)	<0.0001****
Aβ42/40 ratio	0.086 (0.008)	0.041 (0.009)	<0.0001****
P‐tau_181_ (ng/L)	27.8 (11.2)	123.0 (75.6)	0.0030**
T‐tau (ng/L)	291 (86)	783 (449)	0.0085**
NFL (ng/L)	1112 (592)	1702 (820)	0.092
AP2B1 (fmol/µL)	0.59 (0.30)	0.96 (0.4)	0.057
γ‐synuclein (fmol/µL)	0.41 (0.15)	0.61 (0.19)	0.022*
Neurogranin (fmol/µL)	0.027 (0.019)	0.060 (0.032)	0.017*
NPTXR (fmol/µL)	2.32 (1.36)	2.93 (1.11)	0.247
NPTX1 (fmol/µL)	1.36 (1.11)	1.72 (0.80)	0.357
NPTX2 (fmol/µL)	0.72 (0.37)	0.94 (0.33)	0.162
GDI‐1 (fmol/µL)	0.17 (0.063)	0.28 (0.089)	0.0059**
PEBP‐1 (fmol/µL)	14.9 (6.3)	24.4 (7.2)	0.0047**
14‐3‐3 ζ/δ (fmol/µL)	1.24 (0.60)	2.26 (0.69)	0.0018**
Syntaxin‐1B (fmol/µL)	0.10 (0.060)	0.18 (0.079)	0.018*
Syntaxin‐7 (fmol/µL)	0.019 (0.007)	0.025 (0.008)	0.089

*Notes*: Data presented as mean (standard deviation). Analysis of variance (ANOVA) and chi‐squared goodness‐of‐fit test were used to compare continuous and categorical variables between groups. *p ≤0.05, **p ≤0.01, ****p ≤0.0001.

Abbreviations: AP2B1, adaptor‐related protein complex 2 subunit beta 1; AD, Alzheimer's disease; Aβ42/40, amyloid‐β 42/40; CN, cognitively normal; GDI‐1, rab GDP dissociation inhibitor alpha; MMSE, Mini‐Mental State Exam; NFL, neurofilament light; NPTX, neuronal pentraxin; PEBP‐1, phosphatidylethanolamine‐binding protein 1; P‐tau_181_, tau phosphorylated at threonine 181; T‐tau, total tau.

### 3.2 Association between synaptic density and synaptic proteins

**FIGURE 1:**
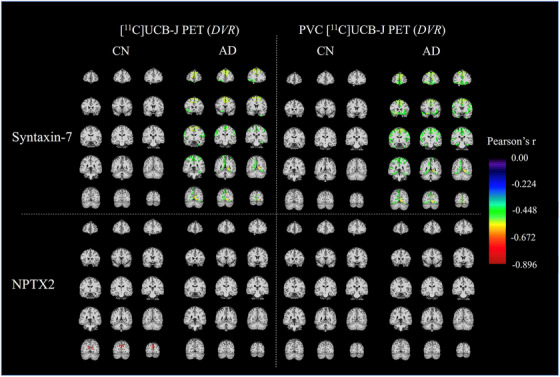
Regional associations (Pearson's *r*) between cerebrospinal fluid syntaxin‐7 or neuronal pentraxin‐2 (NPTX2) and synaptic density (DVR) determined by [11C]UCB‐J PET in Alzheimer's disease (AD) and cognitively normal (CN) participant groups. Analysis was performed with and without partial volume correction (PVC) of PET data.

The primary analysis investigated the association between global synaptic density (*DVR*) in a composite of AD‐affected regions^24^ and synaptic protein levels in the AD group. We also examined the same associations in the the CN group and the total sample (Table S3). In the AD group, significantly higher levels of syntaxin‐7 (*r *= −0.49, *p* = 0.024) and PEBP‐1 (*r *= −0.47, *p* = 0.033) were associated with lower global synaptic density. In the total sample, lower global synaptic density was significantly associated with higher levels of AP2B1, neurogranin, γ‐synuclein, GDI‐1, PEBP‐1, syntaxin‐1B, and syntaxin‐7 (*r *= −0.40 to −0.54, *p* < 0.05) but not with the levels of the neuronal pentraxins or 14‐3‐3 zeta/delta. In the CN group, there were no significant associations between any proteins and global synaptic density. When PVC was performed on PET data, in the AD group, only significantly higher levels of syntaxin‐7 (*r *= −0.51, *p* < 0.05) were associated with lower PVC global synaptic density. In the total sample, lower global synaptic density was significantly associated with higher levels of AP2B1, GDI‐1, and PEBP‐1 (*r *= −0.40 to −0.44, *p* < 0.05). In the CN group, there were no significant associations between any proteins and PVC global synaptic density. When additional analyses assessed the association between CSF protein biomarkers and synaptic density in all brain regions, negative associations were found across many parietal, temporal, prefrontal, and occipital cortical regions (data not shown). The associations varied between biomarkers as seen by syntaxin‐7, which showed the strongest associations with synaptic density in different brain regions, and neuronal pentraxin‐2, which showed no associations with synaptic density as measured (Figure 1). Notably, in the context of AD, where the hippocampus is known to be an area of early synaptic loss, there were no significant associations between any synaptic proteins and hippocampal synaptic density in either the separate groups or total sample (Figure 1 and Table S4).

## 4 DISCUSSION

In this study, we examined the relationship between potential biomarkers of synaptic pathology and synaptic density using a mass spectrometric synaptic protein panel and [^11^C]UCB‐J PET. In the AD group, the most robust global associations were seen for syntaxin‐7 and PEBP‐1, whereas in the total sample, associations were found for several of the synaptic proteins, including the established synaptic biomarker neurogranin. Interestingly, no associations were found for the neuronal pentraxins, synaptic proteins that are emerging as interesting biomarkers in neurodegenerative diseases.

Syntaxin‐7 is involved in vesicle endocytosis at the synapse, as a SNARE protein, and, thus, in the mediation of endocytic trafficking.^26^ Endocytic impairment has been implicated as a feature of many neurodegenerative diseases, not the least in AD.^27^ However, studies of syntaxin‐7 as a potential biomarker have shown no changes in the CSF of AD patients or other diseases compared to controls.^9^, ^25^ We also observed a non‐significant difference between AD patients when compared to controls in the current study. However, syntaxin‐7 was shown to have the strongest association with synaptic density as quantified by [^11^C]UCB‐J PET within AD patients. Since SV2A, the target for [^11^C]UCB‐J, and syntaxin‐7 are both synaptic vesicle proteins the stronger associations between synaptic density and syntaxin‐7 compared to the other synaptic proteins might be attributed to the proximity in function between the two. PEBP‐1 was also associated with [^11^C]UCB‐J PET in AD patients, but displayed higher levels in the AD compared to the CN group. PEBP‐1 is a regulatory protein with modulatory roles in several protein kinase signaling cascades as well as the precursor to the hippocampal cholinergic neurostimulating peptide implicated in the induction of acetylcholine synthesis and enhancement of glutamatergic activity.^28^, ^29^

The neuronal pentraxins are three synaptic proteins, two secreted glycoproteins (NPTX1 and NPTX2), and their plasma membrane‐anchored receptor (NPTXR), which play important roles in synaptic function and plasticity.^30^ By associating and forming heteromultimers, the neuronal pentraxins are involved in the recruitment and localization of neurotransmitter receptors to the post‐synaptic membrane during receptor exocytosis. As previously mentioned, pentraxins have been recognized as potential synaptic biomarkers with decreased levels across neurodegenerative diseases compared to healthy individuals and whose levels strongly correlate with cognitive decline.^9^, ^10^, ^31^, ^32^, ^33^, ^34^, ^35^, ^36^ However, in the current study, neuronal pentraxin levels in the CSF did not differ between AD and CN groups and did not correlate with reduced synaptic density in AD. The cause of this inconsistency is not clear, but differences in AD pathologic stage or other participant characteristics between sample studies could be contributors. This finding in combination with the observation that the levels of the neuronal pentraxins in the CSF of AD patients differ in the directionality of change (decreased instead of increased) compared with most other synaptic proteins might suggest that neuronal pentraxin levels in CSF indicate a unique synaptic pathologic process occurring in AD. Further studies are needed to confirm these findings and to investigate what mechanisms might underlie the difference between synaptic protein alterations in AD.

The main limitation of this study is its modest sample size, which limited its power to detect associations. This might have especially affected the control group, where no associations were seen; nevertheless, all scatter plots (data not shown) were visually inspected, and no convincing associations were observed. However, we are encouraged that several proteins including syntaxin‐7 and PEBP‐1 demonstrated associations with [^11^C]UCB‐J PET in AD. Future studies with larger sample sizes and a broader range of synaptic proteins may lead to a better understanding of the relationship between altered synaptic density fluid synaptic biomarkers in AD.

## 5 CONCLUSION

We observed that the reductions in synaptic density found in AD compared to CN participants using [^11^C]UCB‐J PET are associated with biomarker levels of synaptic proteins.

## AUTHOR CONTRIBUTIONS

Johanna Nilsson, Nicholas J. Ashton, and Adam P. Mecca designed the study. Johanna Nilsson performed the experiments. Johanna Nilsson, Nicholas J. Ashton, Elaheh Salardini, Ryan S. O'Dell, and Adam P. Mecca analyzed the data and wrote the manuscript. Ann Brinkmalm, Henrik Zetterberg, and Kaj Blennow were responsible for supervising, conceptualizating, and verifying the underlying data. Adam P. Mecca, Ryan S. O'Dell, Richard E. Carson, and Christopher H. van Dyck provided the CSF samples of the clinical cohort. Adam P. Mecca, Ryan S. O'Dell, and Christopher H. van Dyck participated in the diagnosis of the patients and CSF sample collection. Ann Brinkmalm, Elaheh Salardini, Richard E. Carson, Christopher H. van Dyck, Henrik Zetterberg, and Kaj Blennow contributed to the interpretation of the results and provided critical feedback of the manuscript. All authors have reviewed the manuscript.

Funders did not have any role in the study design, data collection or analysis, and interpretation of the results or manuscript writing.

This work was supported by the Eivind and Elsa K:son Sylvan Foundation, the Foundation for Gamla Tjänarinnor, and Demensfonden. Henrik Zetterberg is a Wallenberg Scholar supported by grants from the Swedish Research Council (2018‐02532), the European Research Council (681712), Swedish State Support for Clinical Research (ALFGBG‐720931), the Alzheimer Drug Discovery Foundation (ADDF), USA (201809‐2016862), the AD Strategic Fund and the Alzheimer's Association (ADSF‐21‐831376‐C, ADSF‐21‐831381‐C, and ADSF‐21‐831377‐C), the Olav Thon Foundation, the Erling‐Persson Family Foundation, Stiftelsen för Gamla Tjänarinnor, Hjärnfonden, Sweden (FO2019‐0228), the European Union's Horizon 2020 Research and Innovation Programme under the Marie Skłodowska‐Curie grant agreement 860197 (MIRIADE), and the UK Dementia Research Institute at UCL. Kaj Blennow is supported by the Swedish Research Council (2017‐00915), the Alzheimer Drug Discovery Foundation (ADDF), USA (RDAPB‐201809‐2016615), the Swedish Alzheimer Foundation (AF‐742881), Hjärnfonden, Sweden (FO2017‐0243), the Swedish state under the agreement between the Swedish government and the County Councils, the ALF‐agreement (ALFGBG‐715986), the European Union Joint Program for Neurodegenerative Disorders (JPND2019‐466‐236), the National Institutes of Health (NIH), USA (grant 1R01AG068398‐01), and the Alzheimer's Association 2021 Zenith Award (ZEN‐21‐848495). Adam P. Mecca, Ryan S. O'Dell, Richard E. Carson, and Christopher H. van Dyck received support for this work from the National Institute on Aging (P30AG066508, P50AG047270, R01AG052560, R01AG062276).

All participants provided written informed consent as approved by Institutional Human Investigation Committees.

## CONFLICT OF INTEREST STATEMENT

Henrik Zetterberg has served at scientific advisory boards and/or as a consultant for AbbVie, Alector, Annexon, AZTherapies, CogRx, Denali, Eisai, Nervgen, Pinteon Therapeutics, Red Abbey Labs, Roche, Samumed, Siemens Healthineers, Triplet Therapeutics, and Wave, has given lectures in symposia sponsored by Cellectricon, Fujirebio, Alzecure, and Biogen, and is a cofounder of Brain Biomarker Solutions in Gothenburg AB (BBS), which is a part of the GU Ventures Incubator Program. Kaj Blennow has served as a consultant, at advisory boards, or at data monitoring committees for Abcam, Axon, Biogen, and JOMDD/Shimadzu. Julius Clinical, Lilly, MagQu, Novartis, Roche Diagnostics, and Siemens Healthineers and is a cofounder of Brain Biomarker Solutions in Gothenburg AB (BBS), which is a part of the GU Ventures Incubator Program. Adam P. Mecca reports grants for clinical trials from Genentech, Eli Lilly, and Janssen Pharmaceuticals outside the submitted work. Christopher H. van Dyck reports consulting fees from Kyowa Kirin, Roche, Merck, Eli Lilly, and Janssen and grants for clinical trials from Biogen, Novartis, Eli Lilly, Merck, Eisai, Janssen, Roche, Genentech, Toyama, and Biohaven, outside the submitted work. The other authors declare no conflicts of interest. Author disclosures are available in the Supporting Information.

## Supporting information



Supporting Information

Supporting Information

## Data Availability

Derived data supporting the findings of this study are available from the corresponding author on request, provided that data transfer is in agreement with the participating organization's national legislation and Institutional Review Board.
